# Beyond the Biomedical Model: A Critical Review of the Approach to Chronic Pain and the Proposal of an Integrated Functional Model

**DOI:** 10.7759/cureus.85717

**Published:** 2025-06-10

**Authors:** Luigi Pianese, Bruno Bordoni

**Affiliations:** 1 Physical Medicine and Rehabilitation, 3C+A Health and Rehabilitation, Roma, ITA; 2 Physical Medicine and Rehabilitation, Foundation Don Carlo Gnocchi, Milan, ITA

**Keywords:** biomedical model, bio-psycho-social model, chronic headache, fascia, myofascial, pain

## Abstract

Chronic pain represents one of the most complex challenges of contemporary medicine. This narrative review critically analyzes the limitations of the traditional biomedical model in addressing chronic pain, highlighting how its reductionist vision is inadequate to effectively understand and treat this condition. The conceptual evolution of pain from a simple symptom to a codified “disease” is examined, highlighting the incongruities of this approach. The contribution of the bio-psycho-social model is discussed, recognizing its merits but also its applicative limits. Finally, the integration of the functional model, derived from functional medicine, is proposed as a complementary element that could fill the existing gaps, paying greater attention to nutritional and environmental factors and to the mesenchymal matrix as the biological “terrain” of chronic painful diseases. This review suggests the evolution towards a “bio-psycho-functional and social” model for a more complete understanding and management of chronic pain.

## Introduction and background

Chronic pain represents a significant challenge for the contemporary healthcare system, affecting approximately 20% of the global adult population with considerable impacts on quality of life and healthcare costs. Recent epidemiological studies indicate that in Europe, the prevalence of chronic pain reaches 25-30% of the adult population, with direct and indirect costs estimated at approximately 300 billion euros per year [1]. In the United States, chronic pain affects approximately 100 million adults, with an annual economic cost exceeding 635 billion dollars, exceeding the combined costs of cardiovascular disease, cancer, and diabetes [2].

Among the most representative conditions, chronic low back pain affects approximately 7.5% of the world population globally, with a lifetime prevalence reaching 80% [3]. Chronic headache, in its various forms, affects over 15% of the world population, with migraine representing the second leading cause of global disability in the 15-49 age group [4].

The understanding and management of chronic pain have historically been dominated by the biomedical model, a paradigm that, despite its merits, presents significant conceptual and practical limitations, particularly evident in chronic conditions such as persistent low back pain and chronic headache. The different and most of the models to explain and understand pain take into account the sensation of pain coming from electrical, mechanical, thermal, and chemical stimuli.

Despite advances in understanding the neurobiological mechanisms of pain, the clinical approach to this condition often remains unsatisfactory, with many patients not finding adequate relief through conventional treatments [1].

The biomedical model is configured as entirely focused on the treatment of specific physical diseases, putting prevention and the concept of health promotion (health defined as "absence of disease") in the background. It has allowed medicine to make many advances; it must be recognized for the historical advantage of having represented disease as a crucial issue that society must deal with. The biomedical model, developed during the twentieth century, has represented a fundamental pillar in the understanding and treatment of numerous pathologies, showing evident limitations when dealing with complex and multifactorial conditions such as chronic pain. These limitations are manifested not only in the therapeutic efficacy, but also in the conceptualization of pain itself, which oscillates ambiguously between symptoms, alarm, and disease, generating both theoretical and practical confusion [5].

In recent decades, the recognition of the limits of this model has favored the emergence of the bio-psycho-social approach, introduced by Engel in 1977, which integrates biological, psychological, and social factors in the understanding of health and disease [6]. However, this paradigm also has limitations, particularly in its practical application and in the tendency to emphasize psychological aspects more than biological and social ones [7]. This review aims to critically analyze the limits of the biomedical model in the understanding and management of chronic pain, evaluate the contribution and limitations of the bio-psycho-social model, and propose the integration of a functional approach that pays greater attention to nutritional and environmental factors and the mesenchymal matrix as the biological "terrain" of chronic painful pathologies.

## Review

The biomedical model and its limits in the conceptualization of pain

The biomedical model, based on the Cartesian paradigm of mind-body separation and biological reductionism, has represented the theoretical foundation of modern medicine [8]. This approach conceives disease as an alteration of measurable biological parameters, attributable to specific anatomical lesions or identifiable physiological dysfunctions.

In the field of pain, the biomedical model has generated an ambiguous and contradictory conceptualization [9]. For readers who would like to know more about the different facets of the meaning of pain, we recommend you go to the website: International Association for the Study of Pain (IASP). Traditionally, pain has been considered a symptom, or a signal of identifiable tissue damage. This vision, which finds its best-known expression in Descartes's theory of specificity, presupposes a linear relationship between nociceptive stimulus and painful perception [10].

However, clinical experience has repeatedly demonstrated the inadequacy of this concept. In this sense, medical pain management is in crisis. The prevalence of pain is high despite expensive and well-intentioned medical responses, which are mainly based on pharmaceuticals and high-tech interventions. Pain and aspects of current pain management strategies are having enormous deleterious impacts on patients, the healthcare system, and society [11].

Many patients report pain in the absence of identifiable tissue damage, while others with obvious anatomical lesions experience no pain at all. This discrepancy has led to a reformulation of the concept of pain within the biomedical model itself, which has begun to distinguish between pain as an “alarm” (when there is no evident tissue damage), pain as a “symptom” (when the damage is identifiable), and even pain as a “disease”, when it becomes “chronic” (with further sub-categories between “primary chronic pain” and “secondary chronic pain”, in an “endless pathological babel” probably useful only for the further medicalization of pain - our personal reflection), in a tripartite conception of pain that deserves an in-depth critical analysis [12].

This distinction is, however, problematic in several aspects.

Pain as an alarm represents the first conceptual inconsistency of the biomedical model. When pain occurs in the absence of demonstrable tissue damage, it is frequently relegated to the role of a simple alarm signal. This reductionist interpretation considers “pain-alarm” as qualitatively different from “pain-symptom”, ignoring that both share the same subjective experiential nature. As highlighted by the definition of the IASP, pain is “an unpleasant sensory and emotional experience associated with actual or potential tissue damage or described in terms of such damage” [13]. This definition emphasizes the experiential component of pain, regardless of the presence or absence of objective tissue damage. Furthermore, as highlighted by Melzack and Wall since 1965 in their gate theory, the perception of pain necessarily implies a subjective experiential component, making the distinction between pain as an alarm and pain as a symptom artificial [14].

The second problematic manifestation concerns pain as a symptom. According to the traditional biomedical model, pain acquires the status of a symptom in the strict sense only in the presence of an identifiable tissue lesion. This conception arbitrarily excludes phenomena such as social pain, widely documented by psychoneuroendocrinoimmunological (PNEI) research. Eisenberger and Lieberman have demonstrated that social exclusion activates the same brain circuits involved in physical pain, highlighting the real nature of these painful experiences even in the absence of direct anatomical damage [15].

Finally, the most recent evolution of the biomedical model has also led to considering chronic pain as a “disease” in its own right, so much so that it has been included as a specific nosological entity in the International Classification of Diseases (ICD) [12]. This classification, while on the one hand recognizes the complexity and impact of chronic pain, on the other risks promoting a further medicalization of pain, strengthening the pharmacological and interventional approach to the detriment of a broader understanding of the underlying causes that generate it. Recent studies demonstrate how diets rich in pro-inflammatory foods can promote a state of low-grade chronic inflammation, a central element in the physiopathology of persistent pain. Research conducted by Totsch and Sorge has highlighted how certain dietary patterns can modulate the systemic inflammatory response, significantly influencing pain sensitivity and central sensitization mechanisms [16].

The most significant limitation of the biomedical model in the management of chronic pain lies precisely in its inability to adequately consider the primary causes that determine the chronicity of the symptom. Focusing almost exclusively on the neurobiological mechanisms of pain transmission, this approach neglects fundamental factors such as lifestyle, nutrition, and environmental exposure that often result in the basis of the subclinical systemic inflammatory state and that play a crucial role in the onset and maintenance of pain, even chronic pain [17]. The poor correlation between tissue damage and pain perception in many chronic conditions represents an interpretative challenge that the biomedical paradigm struggles to incorporate into its conceptual framework.

The bio-psycho-social model: contributions and limits

The bio-psycho-social model, proposed by George Engel in his article “The Need for a New Medical Model: A Challenge for Biomedicine” published in Science in 1977, represented an attempt to overcome the limitations of the biomedical model, introducing a multidimensional vision of health and disease that integrates biological, psychological, and social factors [6].

This approach has had a significant impact on the understanding of chronic pain, recognizing that the painful experience has a complex and multifactorial nature that is influenced not only by biological processes, but also by psychological factors (such as beliefs, expectations, emotions) and social factors (such as family support, socioeconomic status, cultural context).

The psychological dimension in the bio-psycho-social model has received particular attention, with numerous studies documenting the impact of factors such as catastrophizing, fear of movement, depression, and anxiety on the experience of pain. Flor and Turk have extensively demonstrated how these factors can influence both pain perception and treatment outcomes, justifying the integration of psychological interventions into the management of chronic pain [18]. The social component of the model, although conceptually recognized, has received less empirical attention. Critics of the bio-psycho-social model point to the paucity of research systematically examining the impact of social factors on the pathophysiology of pain, in part due to the methodological difficulties in isolating and quantifying these variables. However, recent studies are beginning to document how factors such as social support, socioeconomic status, and disparities in access to care significantly influence both the incidence and prognosis of chronic pain conditions [19].

The adoption of the bio-psycho-social model has led to important advances in the management of chronic pain, promoting multidisciplinary therapeutic approaches that integrate pharmacological, psychological, and rehabilitative interventions [20]. This model has also contributed to a better understanding of the mechanisms of central and peripheral sensitization, recognizing the role of psychosocial factors in the modulation of the painful experience [10].

However, even the bio-psycho-social model, while representing a significant advancement over the traditional biomedical paradigm, presents important conceptual and practical limitations that deserve in-depth analysis. Despite the inclusion of psychological and social factors, this model continues to pay insufficient attention to crucial determinants of health and, specifically, of the genesis and perpetuation of chronic pain [21].

One of the main gaps of the bio-psycho-social model concerns the marginal consideration of dietary factors in the physiopathology of pain. Contemporary research has widely documented how specific dietary patterns can promote or attenuate systemic inflammatory processes, with direct consequences on nociceptive sensitization and the chronicity of pain [22]. Studies conducted by Totsch and Sorge have highlighted how diets rich in omega-6 fatty acids, refined sugars, and ultra-processed foods promote a chronic pro-inflammatory state that sensitizes the nociceptive system [16]. On the contrary, anti-inflammatory dietary patterns, characterized by high consumption of omega-3 fatty acids, antioxidants, and polyphenols, have demonstrated protective effects against central and peripheral sensitization to pain. The extracellular matrix, a crucial environment for the transmission of nociceptive stimuli, is particularly vulnerable to the effects of systemic inflammation induced by dietary factors. The alteration of the composition and viscoelasticity of the mesenchymal matrix, documented by Langevin et al., represents an important pathogenic mechanism in chronic pain that receives little consideration in the conventional bio-psycho-social model [23].

Furthermore, while the model theoretically recognizes the importance of social factors, in clinical practice and research these aspects are often neglected or considered marginal [24]. As highlighted by critics of the model, there are relatively few studies that thoroughly analyze the impact of social determinants on chronic pain, partly due to the methodological difficulties in conducting large-scale research that adequately considers these variables. The bio-psycho-social model tends to underestimate the impact of lifestyle on the pathophysiology of pain. Physical inactivity promotes structural and functional alterations of connective tissues, resulting in increased fascial stiffness and reduced capacity for absorption and distribution of mechanical forces, central elements in the pathophysiology of painful musculoskeletal conditions such as chronic low back pain [25].

Physical inactivity, in addition to its direct effects on the extracellular matrix, significantly contributes to systemic inflammation through the alteration of metabolic homeostasis and the promotion of pro-inflammatory states [26]. These mechanisms, widely documented in recent scientific literature, receive insufficient attention in the traditional bio-psycho-social model [27].

A particularly significant limitation of the bio-psycho-social model concerns the poor consideration of environmental factors, both in terms of exposure to xenobiotic substances and disconnection from the natural environment [28].

Chronic exposure to anthropogenic physical and chemical factors, such as air pollutants, endocrine disruptors, and artificial electromagnetic fields, has shown significant effects on immune and inflammatory regulation, with potential consequences on pain sensitization [29]. These factors, although representing increasingly relevant determinants in the pathogenesis of chronic inflammatory conditions, receive marginal attention in the conventional bio-psycho-social approach.

Particularly neglected is the lack of “biophysical” contact with the natural environment and its consequences on human health. Emerging research on “earthing” (or “grounding”), summarized by Chevalier et al., documents how direct contact with the Earth’s surface favors the transfer of free electrons to the organism, with significant anti-inflammatory and free radical neutralization effects [30]. The deprivation of this contact, characteristic of the contemporary lifestyle, contributes to the accumulation of reactive oxygen species (ROS) and the maintenance of a chronic pro-inflammatory state, with direct implications in the physiopathology of persistent pain [31].

The combination of these factors (nutritional, behavioral, and environmental) significantly converges on the quality and functionality of the extracellular matrix, a crucial determinant in the physiopathology of chronic pain. The matrix, far from being a simple passive "scaffolding", represents a dynamic environment of cellular communication and modulation of the inflammatory and immune response [32].

The functional model: an approach centered on primary causes

The “functional model”, derived from functional medicine, offers a complementary perspective that could fill the gaps of previous models. This approach focuses on identifying and treating the primary causes of biological dysfunctions, rather than limiting itself to managing symptoms or intervening on the consequences of such dysfunctions [33].

The functional model in understanding health and disease has composite roots, with contributions from different disciplines and research traditions.

The first formal conceptualizations of a functional approach to health can be traced to the work of James Oschman, a cell biologist who in the early 1990s began to develop the concept of the “living matrix” as an integrated communication system [31]. In his article, Oschman proposed a vision of the human body as a continuous communication network, where the extracellular matrix plays a central role in the transmission of biophysical information [31].

Jeffrey Bland coined the term “functional medicine” in the 1990s and founded the Institute for Functional Medicine in 1991, formalizing a clinical approach based on the assessment and optimization of physiological functions rather than the treatment of symptoms [33].

The evolution of the model received further impetus from research on chronic low-grade inflammation and its systemic effects, with significant contributions from Barry Sears and Camillo Ricordi with the “Cellular Inflammation Theory” and David Seaman who explicitly linked nutritional factors, systemic inflammation and chronic pain, laying the foundations for an integrated functional approach [34,35].

Functional medicine considers the organism as a complex and interconnected system, in which the state of health depends on the dynamic balance between different factors: genetic, environmental, nutritional, psychological and social. From this perspective, chronic pain is not simply a symptom to be suppressed or a disease, but the expression of a systemic imbalance that requires targeted intervention on the root causes.

A fundamental concept of the functional model is that of “biological terrain”, represented by the mesenchymal or extracellular matrix, in fact the largest organ of our body, which constitutes the environment in which cells live, communicate, and function. Alterations of this “terrain” can precede and favor the development of pathological processes, including those that lead to peripheral and central sensitization of structures that are part of the pathogenetic mechanisms of pain [36]. The alteration of the matrix, under the combined influence of pro-inflammatory nutritional factors, sedentary lifestyle, and environmental oxidative stress, favors the creation of persistently inflamed tissue microenvironments, contributing significantly to peripheral and central nociceptive sensitization [37]. This pathogenetic mechanism, fundamental in the understanding of chronic pain, receives insufficient attention in the traditional bio-psycho-social model.

The extracellular matrix, a crucial environment for the transmission of nociceptive stimuli, is particularly vulnerable to the effects of systemic inflammation induced by dietary factors. The alteration of the composition and viscoelasticity of the extracellular matrix represents an important pathogenic mechanism in chronic pain that does not receive consideration in the biomedical model, and that also receives little consideration in the conventional bio-psycho-social model [38].

Recent research has also documented how and to what extent the extracellular matrix plays a fundamental role in the development of low-grade inflammation, a central pathogenic condition in numerous chronic pathologies, including those characterized by persistent pain [39]. Some authors have documented how alterations in the composition and architecture of the matrix directly influence the sensitivity and reactivity of peripheral nociceptors through mechanical, biochemical, and electrophysiological mechanisms [40].

Densification and disorganization of the collagen network, documented in several chronic painful conditions, significantly alter the transmission of mechanical forces and the diffusion of inflammatory mediators, contributing to the creation of pro-nociceptive microenvironments characterized by low pH, tissue hypoxia, and accumulation of pro-inflammatory cytokines [41].

The relationship between matrix alterations and mechanisms of central and peripheral pain facilitation is particularly significant. Some authors have highlighted how matrix dysfunctions can favor peripheral sensitization of nociceptors through the up-regulation of ion channels sensitive to mechanical and chemical stimuli, facilitating the transduction of normally non-noxious stimuli into painful impulses [42]. This mechanism, combined with the chronic activation of resident and infiltrating glial cells that characterize matrix alterations, significantly contributes to the transition from acute to chronic pain through the phenomenon of central sensitization [43].

The extracellular matrix also plays a crucial role in regulating immune responses implicated in the pathogenesis of chronic low-grade inflammation. Sorokin's studies have documented how alterations in the molecular composition of the matrix directly influence the recruitment, activation and functional behavior of innate and adaptive immune cells [44]. In particular, the matrix altered by pro-inflammatory stimuli tends to favor the polarization of macrophages towards pro-inflammatory rather than anti-inflammatory phenotypes, perpetuating the chronic inflammatory state that characterizes numerous persistent painful conditions [45].

Of fundamental importance is also the concept of mechanotransduction, i.e., the conversion of mechanical stimuli into biochemical and cellular signals, a process mediated mainly by the extracellular matrix. Langevin et al. have demonstrated how the stiffness and viscoelastic properties of the matrix influence the activation of specific intracellular signaling pathways, which regulate the expression of pro-inflammatory and pro-nociceptive genes [46]. These biomechanical alterations of the matrix, enhanced by pro-inflammatory nutritional factors and sedentary behaviors, represent an important mechanism of amplification and chronification of pain that is systematically neglected in the conventional biomedical and bio-psycho-social model [47].

The extracellular matrix also represents a crucial determinant of the bioavailability and efficacy of inflammatory mediators implicated in nociception. Alterations in the molecular composition of the matrix, in particular the decrease in hyaluronic acid content and the alteration of the sulfation pattern of proteoglycans, significantly influence the diffusion and biological activity of cytokines, chemokines, and growth factors involved in peripheral and central sensitization [48]. This biochemical and biophysical regulation of inflammation, fundamental in the pathogenesis of chronic pain, receives insufficient attention even in the traditional bio-psycho-social model and we can affirm that today the "pathologies of the extracellular matrix" are literally "orphans of diagnosis" also because they are not identifiable and "visible" with traditional imaging models.

Despite the growing scientific evidence on the central role of the extracellular matrix in the pathophysiology of chronic pain, this “biological terrain” remains surprisingly neglected both in research and in clinical practice [49]. The prevalent focus on isolated molecular targets and specific signaling pathways, characteristic of the reductionist approach, has limited the understanding of the systemic complexity of chronic pain and hindered the development of therapeutic interventions aimed at restoring the physiological homeostasis of the matrix [50]. The bio-psycho-social model, while broadening the traditional biomedical vision, continues to pay insufficient attention to this crucial determinant of the pathophysiology of pain, significantly limiting its interpretative and therapeutic efficacy towards chronic painful conditions.

Chronic low-grade inflammation represents one of the main mechanisms through which alterations in the biological terrain influence the perception of pain. This condition, often undetectable through common laboratory tests, can be fueled by multiple factors, including pro-inflammatory diet, disconnection from the natural environment, and exposure to environmental toxins.

Pro-inflammatory Diet

Numerous studies have shown how some food components, such as casein present in dairy products, gluten in cereals, refined sugars, and trans fats, can promote systemic inflammatory processes. These foods can induce alterations in intestinal permeability, dysbiosis of microbiota and immune activation, contributing to the establishment of a chronic inflammatory state that sensitizes the pain pathways [51].

Disconnection from the Natural Environment

Modern life, characterized by constant isolation from the natural environment, i.e., lack of physical contact of the human body with the Earth's surface and with nature in general (using insulating footwear, prolonged stay in closed environments, reduced exposure to natural sunlight), deprives the body of important regulatory and repair mechanisms. Direct contact with the Earth's soil (earthing or grounding) facilitates the transfer of electrons from the Earth to the body, supporting oxidation-reduction processes and neutralization of free radicals, with documented anti-inflammatory effects [29]. Furthermore, it has been demonstrated that grounding the human body can help not only in the prevention of significant symptoms, but also in supporting and assisting recovery from disease. The electrons assimilated from the earth are not only fast-acting antioxidants but also help synchronize the body's hormonal rhythms in contact with the earth's electric field [52].

Exposure to Environmental Toxins 

Air pollutants, heavy metals, pesticides and other environmental contaminants can accumulate in the body, impair cellular function and promote chronic inflammation, contributing to the sensitization of pain mechanisms [53].

All this promotes alterations in mitochondrial function with increased oxidative stress that have been associated with several chronic pain conditions, including fibromyalgia and chronic fatigue syndrome [53].

In this sense, the functional model proposes a therapeutic approach that acts on these primary factors, through targeted interventions, to: modify the diet, eliminating pro-inflammatory foods and introducing anti-inflammatory foods rich in antioxidants, omega-3 fatty acids, and phytonutrients; restore the connection with the natural environment, promoting earthing practices and adequate exposure to sunlight and contact with natural environments; reduce the toxic load through detoxification strategies and minimization of exposure to pollutants; support mitochondrial function and reduce oxidative stress through lifestyle changes; and optimize the quality of sleep, essential for tissue repair and immune regulation processes. Further studies and research are needed to support this postulate.

These interventions aim to modify the “biological terrain” in which chronic pain develops, acting on the primary causes rather than simply suppressing the symptom. Current research, although still limited compared to the vast literature on the biomedical model, has demonstrated the effectiveness of this approach in various chronic pain conditions, from fibromyalgia to rheumatoid arthritis, from headaches to chronic low back pain [54].

Towards an integrated model

The conceptual limitations highlighted suggest the need for an evolution towards a “bio-psycho-functional and social” model that offers a more complete and effective understanding of pain, particularly chronic pain [35]. This integrated paradigm recognizes that pain is not an isolated symptom but a complex experience determined by the interaction of multiple biological, psychological, nutritional, environmental, and social factors [55]. The strength of this model lies precisely in its ability to contextualize pain within a systemic framework, significantly enhancing the effectiveness of therapeutic interventions [56].

This modern vision echoes the pioneering insights of the French physiologist Claude Bernard, who already in the 19th century proposed the revolutionary “terrain theory” (milieu intérieur), according to which the state of health or disease depends primarily on the balance of the internal environment rather than on the sole presence of external pathogens [57]. Bernard argued that “the microbe is nothing, the terrain is everything”, a concept that represents a historical foundation and that apparently found recognition also by his contemporary Louis Pasteur, who on his deathbed admitted: “Bernard was right, the terrain is everything, the microbe is nothing” [58]. This perspective, which further underlines the importance of the internal biological environment in the genesis of diseases, today finds renewed relevance also in the understanding of the physiopathological mechanisms of chronic pain, where the alteration of the “biological terrain”, i.e., the extracellular matrix and the tissue biochemical environment, emerges as a crucial determinant in the transition from acute nociception to persistent pain [59].

The critical analysis of existing models suggests the need for a more comprehensive and integrated approach to chronic pain, which could materialize in a “bio-psycho-functional and social” model. This model would represent an evolution of the traditional bio-psycho-social approach, incorporating a “fourth dimension”, the functional dimension, as a complementary and synergistic element.

Bio-psycho-functional-social model

In this integrated perspective, the biological dimension would include not only the neurobiological mechanisms of pain transmission but also the state of the extracellular matrix, systemic inflammatory processes, and mitochondrial function.

The psychological dimension would continue to consider the impact of cognitive, emotional, and behavioral factors on the pain experience, recognizing the bidirectionality of mind-body interactions.

The functional dimension would emphasize the primary causes of biological dysfunctions, with particular attention to nutrition, environmental exposure, lifestyle, and circadian rhythms.

**Figure 1:**
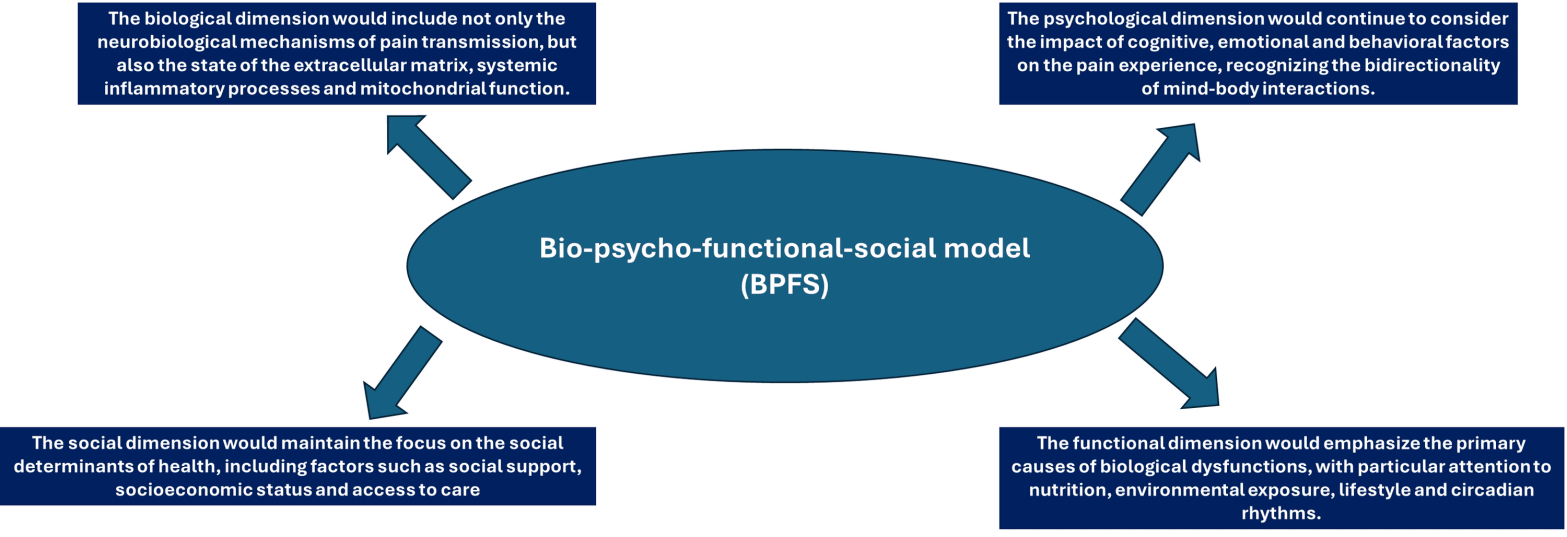
Bio-psycho-functional-social model. The image represents the authors' reflections on how this new model could be based. Figure credit: Luigi Pianese and Bruno Bordoni

The social dimension would maintain the focus on the social determinants of health, including factors such as social support, socioeconomic status, and access to care (Figure 1).

Probably, the integration of these four dimensions would offer a more complete and nuanced understanding of pain (even chronic), recognizing the complexity and uniqueness of each painful experience. This integrated approach would allow us to overcome the false dichotomies that have characterized the debate on pain (organic vs psychogenic, real vs imaginary), recognizing that all pains are “real” and multidimensional. This integrated paradigm recognizes that pain is not an isolated symptom but a complex experience determined by the interaction of multiple biological, psychological, nutritional, environmental, and social factors [60]. The strength of this model lies precisely in its ability to contextualize pain within a systemic framework, significantly enhancing the effectiveness of therapeutic interventions [61].

The bio-psycho-functional-social model conceptualizes chronic pain as a manifestation of a complex biological system in disequilibrium, rather than as a simple consequence of a localized lesion or dysfunction [56]. This integrated view recognizes how factors apparently distant from nociception, such as dietary patterns, environmental exposure, circadian rhythms, and social connection, converge to influence the central pathophysiological mechanisms of persistent pain [19]. Particularly relevant is the understanding of how nutritional factors directly modulate painful experience through inflammatory, oxidative, and neuroendocrine mechanisms [62]. Pro-inflammatory dietary patterns, characterized by high consumption of refined carbohydrates, omega-6 fats, and food additives, promote a state of low-grade systemic inflammation that sensitizes the nociceptive system and favors the transition to chronic pain [63]. Conversely, anti-inflammatory dietary principles have demonstrated efficacy in reducing pain intensity and improving quality of life in several chronic painful conditions, from migraine to fibromyalgia [64].

The functional model also systematically integrates the understanding of how lifestyle and environment influence central and peripheral mechanisms of nociception [65]. Physical inactivity, exposure to environmental pollutants, sleep deprivation, and social isolation are not considered simple aggravating factors but primary determinants of the pathophysiology of chronic pain through their influence on redox balance, mitochondrial activity, and systemic inflammatory processes [66]. This expanded view of pain determinants offers new therapeutic opportunities, overcoming the limitations of conventional approaches mainly focused on analgesic drugs and psychological interventions.

The extracellular matrix as a key mediator in the pathophysiology of pain

In the context of the bio-psycho-functional-social model, the extracellular matrix can be considered not as a primary focus but as a crucial mediator through which the different determinants of health influence the pathophysiological mechanisms of pain [41]. This three-dimensional network of macromolecules represents the biological and biophysical environment in which the integration between environmental, nutritional, behavioral, and psychoemotional stimuli takes place, acting as a “terrain” on which chronic pain processes develop and are maintained.

Matrix alterations, documented in several painful conditions, are not considered simple epiphenomena, but central pathogenetic mechanisms through which systemic factors, such as inflammation, oxidative stress, and mitochondrial dysfunction, translate into peripheral and central nociceptive sensitization [67]. Various studies have shown how alterations in fascial viscoelasticity, mediated by changes in the concentration and structure of hyaluronic acid, directly influence the mechanical sensitivity of musculoskeletal nociceptors, contributing significantly to the pathogenesis of chronic pain syndromes [38].

**Figure 2:**
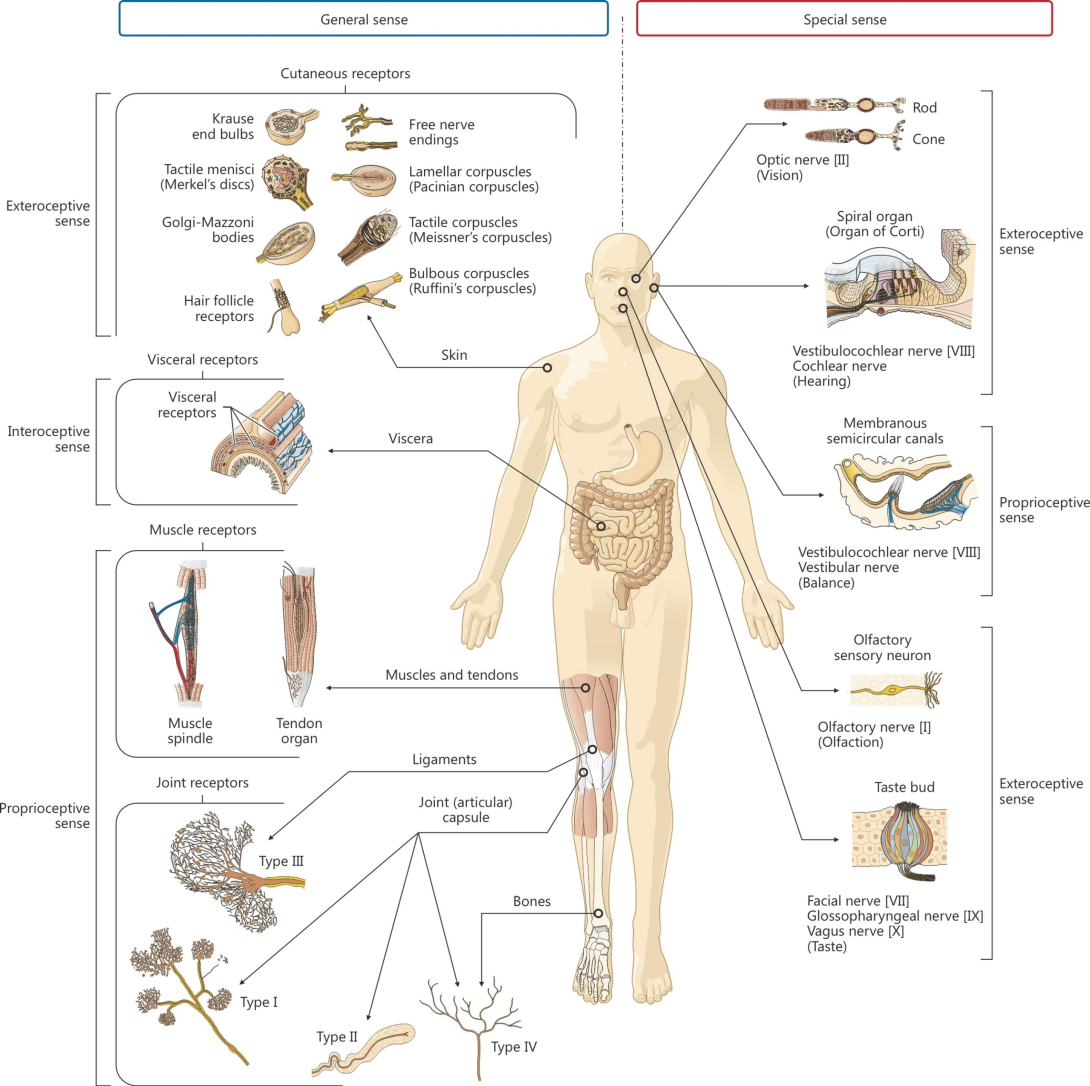
The image shows the richness of multiple different body receptors, including the fascial continuum. Chronic alterations of these receptors can transform innocuous mechanical sensations into nociceptive signals. Reproduced with permission [69]

The fascial system can be a source of pain, not only due to the presence of the passage of nerves to other body structures but also due to the innervation of connective tissue and the presence of mechanical receptors. Free nerve endings contained in the thoracolumbar fascia can send nociceptive information, mimicking a nonspecific lumbar dysfunction; the same post-workout muscle soreness or delayed onset muscle soreness (DOMS) (Figure 2) [68].

Fascial tissue is associated with a solid structure, which can lead to problems related to pain or impaired motor function. Fascia is any tissue that contains features capable of responding to mechanical stimuli. The fascial continuum consists of solid and fluid fascia (blood, lymph, cerebrospinal fluid). The continuum transmits and receives mechano-metabolic information that influences the function of the entire body. A fluid component of the human body is the extracellular matrix which represents approximately 40% of body mass and contains approximately 30% of body proteins. Mechanical stimulation from fluids is crucial for a proper immune response. Lack of movement and adequate communication leads to disease, pain, and inflammation [70].

The functional model also recognizes the extracellular matrix as a privileged therapeutic target for interventions aimed at modulating chronic pain. Manual therapies, myofascial techniques, optimal hydration, and specific nutritional supplementation have demonstrated efficacy in normalizing the biochemical and biomechanical properties of the matrix, with consequent reduction of nociceptive sensitization and improvement of pain perception [71]. These interventions, focused on restoring matrix homeostasis, represent complementary therapeutic tools to conventional pharmacological and psychological approaches, significantly enhancing their efficacy in the treatment of persistent pain.

Clinical implications: multimodal and personalized therapeutic interventions

The adoption of a bio-psycho-functional model translates into a multimodal and personalized therapeutic approach that aims to modify the different factors contributing to the painful experience of the individual patient [11]. Unlike conventional approaches, often characterized by standardized protocols and focused mainly on symptom suppression, the functional model favors interventions calibrated on the specific biochemical, functional, and psychosocial profile of the patient [72].

**Figure 3:**
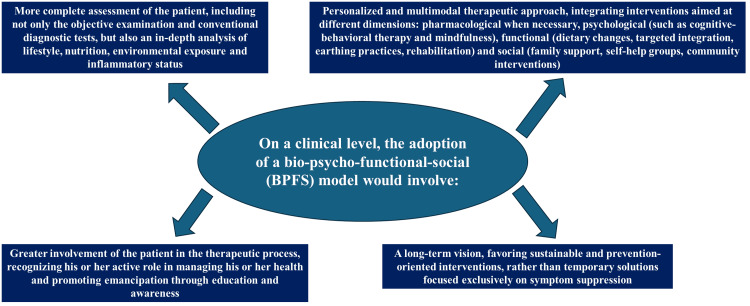
The figure briefly summarizes the advantages of the new model proposed by the authors. Figure credit: Luigi Pianese and Bruno Bordoni

On a clinical level, the adoption of a bio-psycho-functional-social model would involve: a more complete assessment of the patient, including not only the objective examination and conventional diagnostic tests but also an in-depth analysis of lifestyle, nutrition, environmental exposure, and inflammatory status; a personalized and multimodal therapeutic approach, integrating interventions aimed at different dimensions, i.e., pharmacological when necessary, psychological (such as cognitive-behavioral therapy and mindfulness), functional (dietary changes, targeted integration, earthing practices, rehabilitation), and social (family support, self-help groups, community interventions); greater involvement of the patient in the therapeutic process, recognizing his or her active role in managing his or her health and promoting emancipation through education and awareness; a long-term vision, favoring sustainable and prevention-oriented interventions rather than temporary solutions focused exclusively on symptom suppression (Figure 3).

This therapeutic personalization is based on the systematic evaluation of potential physiopathological mediators of pain, including inflammatory status, redox balance, mitochondrial function, microbiome composition, nutritional status, and psychoemotional profile [73]. In this context, the identification of the predominant functional factors in the individual patient guides the selection of targeted interventions that act not only on the symptoms, but on the underlying physiopathological mechanisms, significantly increasing the probability of therapeutic response.

Of relevance is the ability of the functional model to effectively integrate conventional and complementary interventions into a coherent and synergic therapeutic plan [74]. Pharmacological treatments, psychotherapy, manual therapies, nutritional modifications, sleep optimization, and stress management are not considered alternative approaches but complementary components of an integrated therapeutic strategy that simultaneously addresses the different determinants of the painful experience [75]. This holistic vision has demonstrated significantly superior results compared to conventional mono-disciplinary approaches [76].

Evidence supporting the functional model in chronic pain

Although research on the functional model applied to chronic pain is still in its infancy, there is growing evidence supporting the validity of this approach.

Randomized clinical trials conducted on different populations of patients with chronic pain have consistently demonstrated how multimodal interventions based on the principles of the functional model produce significantly superior improvements compared to standard treatments in terms of reduction of pain intensity, functional improvement and quality of life [77]. Particularly relevant are the results obtained in conditions traditionally refractory to conventional approaches such as fibromyalgia and chronic fatigue syndrome [78]. A study conducted by Kaplan et al. documented how an integrated protocol that combined anti-inflammatory nutritional modifications, targeted supplementation, sleep optimization, and stress reduction techniques produced a very high average reduction in pain intensity in patients with fibromyalgia, compared to that observed in the control group treated with the standard protocol [79].

Existing research in systems biology also provides mechanistic support to the functional model, documenting the complex interactions between nutrition, microbiome, immune system, and nociceptive signaling. Cross-sectional studies have highlighted significant correlations between systemic inflammation markers, impaired intestinal integrity and pain intensity in several chronic conditions, supporting the concept of a “gut-brain axis” as a key mediator in the pathophysiology of persistent pain [80]. In the context of chronic low back pain, the functional approach provides a complementary perspective to the bio-psycho-social model. Some authors have documented how patients with chronic low back pain frequently present alterations of the intestinal microbiota, with reduced bacterial diversity and increased pro-inflammatory strains. This intestinal dysbiosis has been associated with elevated levels of systemic inflammatory markers, suggesting a mechanism through which functional alterations of the intestinal ecosystem may contribute to the maintenance of chronic low back pain [81].

Similarly, it has been observed that there are profound relationships between the functional and biomechanical habits of individuals and the possibility of developing nonspecific low back pain even in healthcare settings. Some authors have observed that a sedentary lifestyle has significantly increased the incidence of recurrent and nonspecific low back pain (over 40%) in nursing, health, and medical professions. Within the group of individuals with a sedentary lifestyle, a significant effect of the components of the metabolic syndrome and excessive coffee consumption on the increased probability of nonspecific low back pain was found [82].

From the matrix point of view, alterations of the thoracolumbar fascia can significantly contribute to the genesis and maintenance of chronic low back pain, suggesting that the trend of the thickness of the thoracolumbar fascia indicates an altered fascial remodeling compared to healthy subjects, as a sort of "frozen back". The fascial tissue, a dynamic organ rich in nociceptors and mechanoreceptors, can perpetuate chronic painful states independently of the structures most considered in the traditional biomedical model [83].

In the context of chronic headache, the functional approach offers important interpretative and therapeutic insights. Recent literature has highlighted the role of neurogenic inflammation and central sensitization in the pathogenesis of migraine and other forms of chronic headache. Ramsden et al. have documented how changes in the composition of dietary fatty acids, in particular the reduction of pro-inflammatory omega-6 fatty acids and the increase of anti-inflammatory omega-3, can significantly reduce the frequency and intensity of migraine attacks, suggesting a direct effect of nutrition on the inflammatory mechanisms involved in headache [62].

From the matrix point of view, studies conducted by Fernández-de-las-Peñas et al. revealed significant alterations in the craniocervical fascial tissue in patients with chronic tension-type headache, characterized by increased stiffness and reduced elasticity. These changes, detectable through elastography techniques, correlate with the frequency and intensity of headache episodes, suggesting a causal or contributory role of fascial dysfunctions in the pathogenesis of headache [84].

Emerging evidence on the role of intestinal dysbiosis in the pathogenesis of chronic pain is particularly interesting, with studies documenting significant alterations in the composition of the microbiome in patients with fibromyalgia, irritable bowel syndrome and chronic pelvic pain [85]. Targeted interventions to restore intestinal eubiosis, through dietary modifications, selected probiotics and support for intestinal barrier function, have shown promising results in modulating systemic inflammation and reducing pain intensity in these conditions [86].

The growing body of evidence supporting the clinical efficacy of the functional model is further strengthened by advances in understanding the epigenetic mechanisms that mediate the influence of environmental and nutritional factors on the expression of genes involved in pain regulation. Nutrigenomic literature has documented how specific food components modulate the expression of pro- and anti-inflammatory genes through epigenetic mechanisms, providing a biological substrate for the clinically observed correlation between dietary patterns and pain intensity in chronic conditions [87].

This evidence, although preliminary in some areas, suggests that the integration of a functional approach in the management of chronic pain may offer significant benefits, particularly in patients who have not found adequate relief through conventional treatments.

Implications for research

The adoption of the bio-psycho-functional-social model has profound implications for clinical practice and research in the field of chronic pain, suggesting the need for a fundamental rethinking of the diagnostic and therapeutic approach to these conditions [21]. On the clinical level, this model requires the implementation of multifactorial assessment protocols that systematically investigate the different physiopathological mediators potentially contributing to the pain experience of the individual patient [88].

Unlike the conventional approach, often focused on the search for isolated structural lesions or biochemical alterations, the functional model promotes a systemic assessment that includes analysis of nutritional status, inflammatory profile, mitochondrial function, microbiome composition, and oxidative status [89]. Depending on the medical specialty, there are instrumental investigation and diagnostic evaluation procedures to clinically frame a patient. The clinician's view of the patient may depend on their specialty. This multifactorial characterization allows the identification of specific physiopathological patterns that guide the selection of personalized therapeutic interventions, significantly increasing the probability of clinical response [90].

On the therapeutic level, the functional model promotes the synergistic integration of conventional and complementary interventions in a coherent and personalized treatment plan [91]. This approach requires the development of interdisciplinary teams that include not only physicians and psychologists but also nutritionists, physiotherapists, experts in environmental medicine, and other professionals with specific expertise in the different functional domains relevant to chronic pain [92].

For future research, the bio-psycho-functional-social model suggests the need to overcome traditional monofactorial study designs, favoring approaches that simultaneously investigate the impact of multimodal and personalized interventions. This requires the development of new methodological frameworks that integrate quantitative and qualitative approaches, complex systems analysis and personalized medicine strategies [93].

Particular attention should be paid to research on patient stratification based on specific physiopathological profiles, to identify subgroups that may respond differently to targeted interventions. This “functional phenotyping” approach could overcome the heterogeneity that characterizes many chronic painful conditions, significantly improving diagnostic accuracy and therapeutic efficacy [94].

Finally, the implementation of the functional model in daily clinical practice requires the development of targeted educational programs for health professionals, traditionally trained according to the biomedical paradigm [95]. This cultural change, although complex, represents a necessary step towards a more effective and compassionate approach to chronic pain, which recognizes the multifactorial complexity of these conditions and values ​​the active role of the patient in their healing journey [96]. From a neurobiological point of view, we know that there are several spinal and cerebral areas that process what will become nociceptive information, and this leads us to further confirm the need to think and obtain a new model of pain to better understand how to help the patient in daily clinical practice [97].

## Conclusions

Chronic pain represents a complex challenge that requires an equally complex and multidimensional approach. The traditional biomedical model, with its reductionist vision and its ambiguous conceptualization of pain, has shown clear limitations in the understanding and management of this condition. The bio-psycho-social model has represented an important step forward, but in its practical application it often tends to overlook crucial factors related to nutrition, environment, and lifestyle that influence the state of the extracellular matrix.

Overcoming the limitations of the biomedical model and enhancing the insights of the bio-psycho-social model can be achieved by integrating the “three dimensions” of the “bio-psycho-social” model with a “fourth dimension”: the functional one. We propose a bio-psycho-functional-social model, which embraces the complexity of chronic pain in all its facets. The integration of the functional model, with its focus on the primary causes of biological dysfunctions and the “mesenchymal terrain” in which pathological processes develop, offers a complementary perspective that could fill the existing gaps. An integrated bio-psycho-functional-social model would represent a more comprehensive and personalized approach to chronic pain, while recognizing the complexity and uniqueness of each painful experience.

Evidence supporting this integrated approach is growing, suggesting that interventions aimed at modifying the “biological terrain” through dietary changes, reduction of exposure to environmental toxins, earthing practices, and support of mitochondrial function can offer significant benefits in several chronic pain conditions.

Adopting this integrated model would require significant changes in the training of healthcare professionals, organization of services, and allocation of resources. However, the potential benefits in terms of therapeutic efficacy, quality of life of patients, and economic sustainability of healthcare systems largely justify these efforts.

In conclusion, it is time to overcome partial and reductionist visions of chronic pain, recognizing the complexity of this condition and the need for a truly integrated approach that considers all dimensions of human experience: biological, psychological, functional, and social. Only through this holistic vision will it be possible to offer adequate responses to the suffering of millions of people affected by chronic pain, promoting not only symptom relief, but an authentic recovery of health and well-being. Recognizing and addressing the primary causes of chronic pain represents a decisive step towards a more complete and effective understanding of pain, promoting a truly holistic and personalized therapeutic approach.
